# Association between serum lipase levels and chronic kidney disease stages

**DOI:** 10.1371/journal.pone.0330329

**Published:** 2025-08-18

**Authors:** Gui Zhang, Zuojie Li, Cheng Xin Xu

**Affiliations:** 1 Department of Clinical Laboratory, Taizhou Hospital Shanghai University Traditional Chinese Medicine Taizhou City Traditional Chinese Medicine Hospital, Taizhou, Zhejiang Province, China; 2 Department of Clinical Laboratory, The People’s Hospital of Cangnan Zhejiang, Wenzhou, Zhejiang Province, China; 3 Department of Clinical Laboratory, Linhai Second People’s Hospital, Linhai, Zhejiang, Province, China; The University of the West Indies, JAMAICA

## Abstract

**Objective:**

To explore the relationship between serum lipase levels and chronic kidney disease (CKD) progression, evaluating its usefulness in assessing efficacy, severity, and predicting outcomes in CKD.

**Methods:**

This retrospective study analyzed 251 CKD patients treated at our hospital from January 2018 to December 2023, categorizing them into four groups based on 2022 Chinese and 2024 KDIGO guidelines. Clinical and biochemical data, including serum lipase, Scr, and BUN were collected. Additionally, a supplementary cohort of 50 CKD patients treated between January 2024 and March 2025 was included to support the findings.

**Results:**

Serum lipase levels increased with advancing CKD stages [G1-2: 40.50 (30.75,52.00), G3:47.00 (37.25,61.00), G4:100.00 (77.00,126.00), G5:155.50 (126.75,243.75), (P < 0.05), showing a positive correlation with Scr (r = 0.714, P < 0.001) and BUN ((r = 0.678, P < 0.001). Univariate logistic regression analysis indicated that age, Scr, BUN, and serum lipase were positively associated with CKD stages, whereas HDL-C exhibited negative correlations. Multivariate logistic regression analysis identified several associated factors of CKD stages, including Scr (OR=1.01; 95% CI 1.01–1.02; P < 0.001), BUN (OR=1.17; 95% CI 1.07–1.29; P < 0.001), age (OR=1.03; 95% CI 1.01–1.05; P = 0.019), and serum lipase (OR=1.02, 95% CI 1.01–1.03, P < 0.001). Serum lipase’s AUC for distinguishing CKD stages G1-2 vs. G3, G3 vs. G4, and G4 vs. G5 were 0.64 (0.55–0.73), 0.89 (0.83–0.94), and 0.84 (0.75–0.93), respectively, with validation cohort AUCs of 0.65 (0.45–0.85), 0.82 (0.62–1.00), and 0.86 (0.65–1.00).

**Conclusions:**

Serum lipase emerges as a novel biomarker for CKD stages, exhibiting stage-dependent increases and independent prognostic significance. Regular testing could improve risk assessment and complement current markers like eGFR and proteinuria, enhancing CKD management.

## 1. Introduction

Chronic kidney disease (CKD) affects an estimated 10–12% of the global population, representing a significant public health concern [1]. CKD is characterized by damage to the renal tubules, blood vessels, and interstitium, often accompanied by pathological processes such as inflammation, fibrosis, and hyperfiltration [2]. Due to its asymptomatic nature in the early stages, CKD is typically diagnosed through the detection of proteinuria via urinalysis or elevated creatinine levels in blood tests [3]. As CKD advances, patients may develop complications, including cardiovascular diseases, metabolic disorders, and abnormalities in bone mineral metabolism, all of which can substantially affect their quality of life and overall prognosis [4,5]. The monitoring of biochemical markers is essential for evaluating disease progression and outcomes in the management of chronic kidney disease (CKD). While traditional lipid markers, such as low-density lipoprotein (LDL-C) and high-density lipoprotein (HDL-C), have been extensively investigated, serum lipase warrants particular attention due to its dual role in lipid metabolism and inflammatory regulation—two critical pathways involved in the pathogenesis of CKD. This underscores the importance of examining serum lipase expression profiles in the diagnosis and management of CKD. Serum lipase serves as a significant biomarker, with variations in its levels potentially indicating changes in renal function and their implications for overall metabolic health. Studies have demonstrated a positive correlation between serum lipase levels and the severity of renal impairment in CKD patients [6]. Elevated lipase levels may indicate an increased cardiovascular risk, highlighting their significance in clinical monitoring. Existing research indicates that serum lipase levels increase with the severity of CKD, reflecting metabolic dysregulation and potential organ damage [7]. Recent studies suggest that pancreatic disorders, obesity, and acute kidney injury (AKI) may significantly influence serum lipase levels in individuals with chronic kidney disease (CKD). Notably, AKI has been linked to elevated pancreatic-specific lipase activity, potentially due to reduced lipase clearance as a consequence of impaired renal function [8]. Furthermore, obesity, a prevalent metabolic disorder, may exacerbate CKD progression through inflammatory pathways and adipose tissue dysfunction. Mechanistically, obesity can increase oxidative stress and inflammatory responses, thereby affecting the progression of both AKI and CKD and contributing to alterations in lipase levels [9]. A comprehensive understanding of these confounding factors and their impact on serum lipase is essential for the development of personalized management strategies for CKD patients.

We propose that serum lipase levels exhibit a positive correlation with the stages of chronic kidney disease (CKD) and may serve as an indicator of disease severity. In acknowledging potential confounding factors such as pancreatic dysfunction and acute kidney injury (AKI), this study employed stringent exclusion criteria to eliminate patients with acute pancreatitis, malignancies, or AKI. The rationale for emphasizing serum lipase over other lipid markers lies in its unique capacity to concurrently reflect metabolic disturbances and subclinical inflammation, which are integral to the pathophysiology of CKD stages. Recent studies have shown that in individuals with CKD, both the mass and enzymatic activity of lipoprotein-associated phospholipase A2 (Lp-PLA2) are significantly elevated and positively correlated with low-density lipoprotein (LDL-C) levels. The increased presence of this enzyme is potentially associated with inflammatory processes and the pathogenesis of atherosclerosis, thereby contributing to renal impairment and the exacerbation of cardiovascular disease [10]. Furthermore, disorders of lipid metabolism in patients with chronic kidney disease (CKD) may exacerbate the deterioration of renal function. In individuals diagnosed with systemic lupus erythematosus (SLE), empirical findings reveal significantly elevated serum concentrations of triglycerides (TG), total cholesterol (TC), low-density lipoprotein (LDL-C), and apolipoprotein B (ApoB), alongside reduced levels of high-density lipoprotein (HDL-C) and apolipoprotein A1 (ApoA1). The severity of these lipid metabolism disorders is closely correlated with increased disease activity and renal dysfunction, highlighting a significant association between lipid metabolic disturbances and renal function parameters [11]. Moreover, certain studies suggest that interventions aimed at modulating lipid metabolism may improve the overall prognosis for patients with CKD. Therefore, the monitoring of serum lipase levels may prove to be a valuable tool in assessing disease progression and guiding personalized treatment approaches [12].

Serum lipase, recognized as a pivotal biomarker, may contribute to the exacerbation of renal function deterioration through its interactions with lipid metabolism disorders, inflammation, and alterations in the immune system. This study seeks to investigate the clinical utility of serum lipase in evaluating disease progression and prognosis in chronic kidney disease (CKD), with a particular focus on its independence from traditional renal biomarkers and its association with multisystem complications. The findings aim to contribute novel strategies for diagnosis and treatment.

## 2. Materials and methods

### 2.1 Study participants

The present study received approval from the Ethics Committee of Taizhou Hospital, affiliated with Shanghai University of Traditional Chinese Medicine and Taizhou City Traditional Chinese Medicine Hospital (Approval Nos. LL2024-LW-045 and LL2025-LW-020). The research was conducted in compliance with the principles outlined in the Declaration of Helsinki. This investigation involved a retrospective review of patient case records diagnosed with chronic kidney disease (CKD). Data collection occurred from April 1 to April 7, 2025, utilizing the hospital information system (HIS), electronic medical records (EMR), and the laboratory information management system (LIS). Initially, a cohort of 1,562 patients with CKD, who received treatment at our institution between January 2018 and December 2023, was considered for inclusion. The inclusion criteria were defined as follows: (1) a clinical diagnosis aligning with the Early Screening, Diagnosis, and Prevention Guidelines for Chronic Kidney Disease (2022 Edition) and the KDIGO 2024 Clinical Practice Guideline for the Evaluation and Management of Chronic Kidney Disease [13,14]; The study included participants aged 18–80 years. The exclusion criteria were as follows: (1) current participation in renal replacement therapy; (2) the presence of severe respiratory, digestive, or hematologic diseases, as well as acute or severe infections or malignancies; (3) autoimmune diseases; (4) acute diabetic complications, such as diabetic ketoacidosis or hyperosmolar hyperglycemic coma; (5) a history of chronic alcoholism, defined as consumption of more than 14 drinks per week for males or more than 7 drinks per week for females, or macro-lipasemia, characterized by serum lipase levels exceeding three times the upper limit of normal without pancreatic pathology; and (6) incomplete essential data, including serum creatinine levels and urine albumin-to-creatinine ratio (UACR). All participants underwent lipase testing and liver function assessments (ALT, AST, and bilirubin) at the time of enrollment. Abnormal results prompted diagnostic imaging, such as abdominal ultrasound or CT, to exclude subclinical pancreatic or hepatic pathologies. Ultimately, 251 patients met the eligibility criteria and were included in the study, comprising 174 males and 77 females. Furthermore, an auxiliary cohort comprising 50 patients diagnosed with chronic kidney disease (CKD) (40 males, 10 females), who met the same criteria, were recruited between January 2024 and March 2025 to facilitate external validation.

### 2.2 Diagnosis and stages of CKD

This study complies with the 2022 guidelines established by China and the 2024 KDIGO guidelines [13,14]. Patients with chronic kidney disease (CKD) were classified into six groups according to estimated glomerular filtration rate (eGFR) criteria: stage 1 (eGFR ≥ 90 ml/min/1.73 m²), stage 2 (eGFR 60–89 ml/min/1.73 m²), stage 3a (eGFR 45–59 ml/min/1.73 m²), stage 3b (eGFR 30–44 ml/min/1.73 m²), stage 4 (eGFR 15–29 ml/min/1.73 m²), and stage 5 (eGFR < 15 ml/min/1.73 m²). As the majority of patients had proteinuria assessed only once, persistent proteinuria was not defined in this study. Additionally, due to the limited sample size, stages G1 and G2 were consolidated into G1–2, and stages G3a and G3b were combined into G3.

### 2.3 Clinical and biochemical indexes

At the time of enrollment, patients diagnosed with hypertension and diabetes were classified based on established criteria. Hypertension was defined as a systolic blood pressure of 140 mmHg or higher, a diastolic blood pressure of 90 mmHg or higher, or a documented history of antihypertensive treatment [15]. In contrast, diabetes was identified through a fasting plasma glucose level of 7.0 mmol/L or higher [16], the use of hypoglycemic medications, or the administration of parenteral insulin therapy. The body mass index (BMI) was calculated using the formula: BMI = weight (kg)/ [height (m)]². Peripheral venous blood and urine samples were collected in a fasting state prior to admission for clinical treatment. Biochemical parameters, including serum lipase, urinary microalbumin, blood lipids, and renal function, were evaluated using the Beckman AU 5800 automatic biochemical analyzer. Specimens exhibiting severe lipemia underwent high-speed centrifugation at 12,000 × g for 15 minutes to isolate serum or plasma, thereby reducing turbidity interference.

### 2.4 Statistical analyses

The data were analyzed using SPSS version 26.0, and graphical representations were generated with GraphPad Prism version 10.0. Continuous variables following a normal distribution are presented as mean ± standard deviation, whereas skewed continuous variables are reported as median (interquartile range). Categorical variables are expressed as count (percentage). Group differences were evaluated using the χ² test for categorical variables and either the Wilcoxon rank-sum test or the t-test for continuous variables. Spearman’s correlation test was applied for correlation analysis, and logistic regression analysis was conducted to express effect sizes as odds ratios (ORs) with their corresponding 95% confidence intervals (95% CIs). The sample size was determined using PASS version 15.0 software, based on preliminary LPS data: G 1–2: 40.3 ± 14.3 U/L, G 3: 53.4 ± 28.3 U/L, G 4: 86.6 ± 48.1 U/L, and G 5: 117.7 ± 78.5 U/L. With a significance level (α) of 0.05 and a power of 0.9, and assuming equal allocation, the formula for multiple means comparison indicated a requirement of 26 participants per group. To accommodate a 20% attrition rate, the minimum required sample size was adjusted to 33 participants per group, resulting in a total sample size of at least 132 participants. Statistical analyses were conducted using R software, version 4.2.0. The analyses encompassed logistic regression, forest plot generation, and receiver operating characteristic (ROC) curve analysis, all performed within the R environment. Model predictor selection was guided by clinical relevance and the outcomes of univariate analyses, with a significance threshold set at P < 0.05. To mitigate multicollinearity, serum creatinine and blood urea nitrogen were incorporated into the final model in place of the estimated glomerular filtration rate (eGFR), and collinearity among variables was evaluated using variance inflation factor (VIF) analysis, with a VIF value below 5 considered acceptable. The model’s discriminative capacity was assessed through ROC curve analysis, which included calculation of the area under the curve (AUC), as well as measures of accuracy, sensitivity, specificity, positive predictive value (PPV), and negative predictive value (NPV).

## 3. Results

### 3.1 Baseline characteristics

In a cohort study involving 251 patients diagnosed with chronic kidney disease, the median age was determined to be 57 years, with participants ranging from 18 to 80 years of age. The sample population consisted of 174 males (69.32%) and 77 females (30.68%). A comparative analysis of baseline characteristics among the four groups revealed statistically significant differences in several parameters, including age, body mass index (BMI), diastolic blood pressure, high-density lipoprotein cholesterol (HDL-C), serum creatinine (Scr), blood urea nitrogen (BUN), estimated glomerular filtration rate (eGFR), and serum lipase (P < 0.05). In contrast, no significant differences were identified in terms of gender, systolic blood pressure, history of diabetes, total cholesterol (TC), triglycerides (TG), low-density lipoprotein cholesterol (LDL-C), uric acid(UA), or urinary albumin levels (P > 0.05), as presented in Table 1.

**Table 1:** Baseline characteristics of the January 2018 to December 2023 cohort.

Characteristics			Overall^1^	Patients with chronic kidney disease (CKD)				Statistics	P value^#^
				Stage 1–2^†^	Stage 3^†^	Stage 4^†^	Stage 5^†^		
Sample size			251	52	126	37	36	—	—
Age (years)			57.00 [49.00, 67.00]	51.50 [42.75, 58.25]	58.00 [51.00, 68.00]	61.00 [50.00, 68.00]	58.00 [50.00, 70.25]	16.014	0.001
Gender	Male		174 (69.32%)	35 (67.31%)	86 (68.25%)	28 (75.68%)	25 (69.44%)	0.869	0.833
	Female		77 (30.68%)	17 (32.69%)	40 (31.75%)	9 (24.32%)	11 (30.56%)		
BMI (kg/m^2^)			21.65 [20.21, 23.58]	21.31 [19.65, 22.93]	22.41 [20.71, 23.69]	21.56 [19.63, 22.77]	21.45 [19.67, 22.66]	10.314	0.016
SBP (mmHg)			132.00 [125.00, 139.00]	130.00 [125.00, 137.75]	132.00 [127.00, 136.00]	128.00 [123.00, 140.00]	130.00 [120.00, 148.75]	2.161	0.540
DBP (mmHg)			81.00 [75.00, 86.00]	80.00 [75.75, 85.00]	83.00 [80.00, 86.00]	75.00 [74.00, 83.00]	75.00 [70.00, 88.50]	16.417	<0.001
Diabetes		Yes	89 (35.46%)	15 (28.85%)	46 (36.51%)	15 (40.54%)	13 (36.11%)	1.478	0.687
		None	162 (64.54%)	37 (71.15%)	80 (63.49%)	22 (59.46%)	23 (63.89%)		
TC (mmol/L)			4.59 [3.87, 5.39]	4.44 [3.89, 5.27]	4.67 [3.90, 5.41]	4.97 [3.99, 5.61]	4.18 [3.61, 5.00]	5.506	0.138
TG (mmol/L)			1.68 [1.11, 2.41]	1.71 [1.07, 2.50]	1.55 [1.02, 2.43]	1.80 [1.34, 2.08]	1.73 [1.20, 2.74]	2.014	0.570
HDL-C (mmol/L)			1.09 [0.94, 1.27]	1.13 [1.00, 1.32]	1.13 [0.97, 1.30]	1.04 [0.96, 1.25]	0.92 [0.73, 1.10]	16.712	<0.001
LDL-C (mmol/L)			2.76 [2.12, 3.48]	2.61 [2.19, 3.36]	2.81 [2.11, 3.47]	3.09 [2.37, 3.86]	2.46 [1.90, 3.36]	5.090	0.165
Urinary protein (mg/L)			445.00 [80.20, 1,136.65]	318.95 [91.75, 913.75]	482.55 [69.98, 1293.50]	549.70 [123.90, 1416.00]	486.45 [141.90, 1018.23]	0.846	0.838
Scr (μmol/L)			124.00 [98.00, 212.00]	85.50 [73.75,105.50])	116.00 [100.50,144.75]	233.00 (173.00,299.00)	413.50 [252.50,562.25]	158.913	<0.001
UA (μmol/L)			415.68 (113.67)	402.38 (91.17)	418.54 (108.10)	439.49 (120.28)	400.42 (149.78)	2.026	0.567
BUN (mmol/L)			9.13 [6.74, 14.93]	6.17 [5.07,7.58]	8.28 [6.72,10.65]	16.06 [12.40,18.19]	21.23 [17.043,29.733]	138.003	<0.001
eGFR (ml/min/1.73 m^2^)			52.93 [27.25, 70.03]	81.84 [64.94,101.36]	55.98 [43.29,66.86]	25.89 [18.94,33.56]	11.32 [8.85,17.82]	160.226	<0.001
Serum lipase (U/L)			53.00 [39.00, 92.00]	40.50 [30.75,52.00]	47.00 [37.25,61.00]	100.00 [77.00,126.00]	155.50 [126.75,243.75]	137.176	<0.001

**Note:** ^†^– Median [M1, M3]; n (%); Mean (SD); ^#^ –Kruskal-Wallis rank sum test; Pearson’s Chi-squared test; BMI – body mass index; SBP –systolic blood pressure; DBP –diastolic blood pressure; TC – total cholesterol; TG – triglycerides; LDL-C – low-density lipoprotein cholesterol; HDL-C – high-density lipoprotein cholesterol; Scr – serum creatinine; UA – uric acid; BUN – blood urea nitrogen; eGFR –estimated glomerular filtration rate.

### 3.2 Serum lipase levels in patients with chronic kidney disease

**Fig 1:**
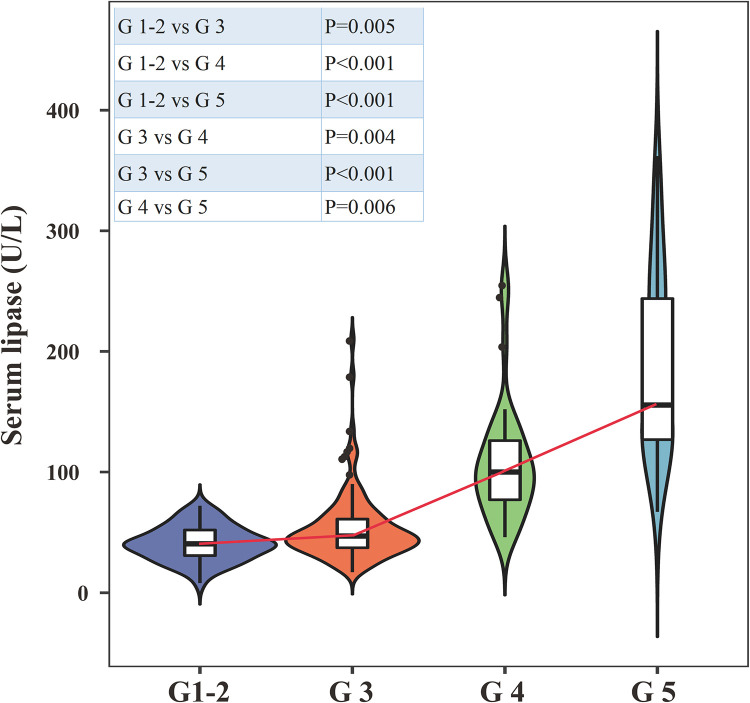
Serum lipase levels across chronic kidney disease stages (Violin plots illustrate serum lipase levels (U/L) across CKD stages G1–2, G3, G4, and G5, showing data distribution, median (horizontal line), and interquartile range (box). Pairwise comparisons used the Kruskal-Wallis test and Dunn’s multiple comparisons, with significant p-values noted in the table (e.g., G1–2 vs G4: P < 0.001).

Serum lipase levels exhibited significant differences across various stages of chronic kidney disease (CKD) (P < 0.05), demonstrating a progressive increase as the stages advanced (Fig 1).

### 3.3 Correlation analysis between serum lipase and renal function indicators (Scr and BUN)

**Fig 2:**
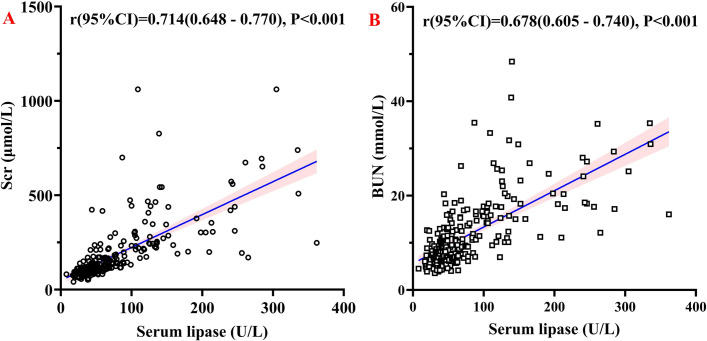
Correlation analyses of serum lipase with renal function parameters (Scr and BUN). Note: A: Scatter plot of serum lipase against serum creatinine (Scr); B: Scatter plot of serum lipase against blood urea nitrogen (BUN. Solid lines show linear regressions with shaded 95% confidence intervals. Each panel displays Pearson correlation coefficients (r) and p-values..

The analysis identified positive correlations in patients with chronic kidney disease: serum lipase levels were positively correlated with serum creatinine (r = 0.714, P < 0.001) and blood urea nitrogen (r = 0.678, P < 0.001), as illustrated in Fig 2.

### 3.4 Analysis of CKD-related risk factors

**Fig 3:**
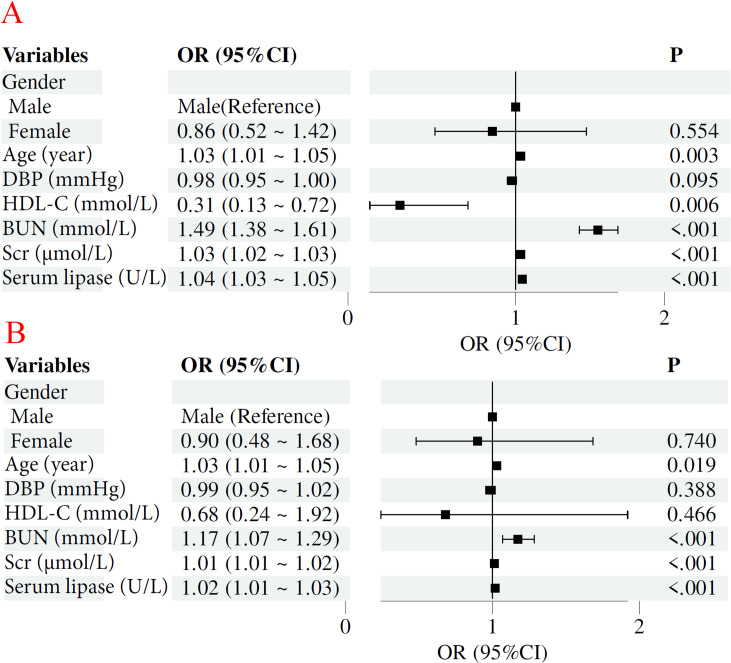
Forest plot of the univariate and multivariate Logistics regression analyses in CKD of the 2018 January to December 2023 cohort. Note: A: Univariate logistic regression forest plot displaying odds ratios (OR), 95% confidence intervals (CI), and p-values for variables such as age, Gender, DBP, HDL-C, Scr, BUN, and serum lipase. B: Multivariate logistic regression forest plot for the same variables, including OR, 95% CI, and p-values, focused on CKD stage progression. Models were adjusted for relevant clinical factors.

Univariate logistic regression analysis identified age, serum creatinine (Scr), blood urea nitrogen (BUN), and serum lipase as positive associated factors of chronic kidney disease (CKD) progression, whereas high-density lipoprotein cholesterol (HDL-C) was were negatively correlated. Multivariate logistic regression analysis further confirmed significant associations with CKD stages for Scr (OR=1.01; 95% CI 1.01–1.02; P < 0.001), BUN (OR=1.17; 95% CI 1.07–1.29; P < 0.001), Age (OR=1.03; 95% CI 1.01–1.05; P = 0.019), and serum lipase (OR=1.02, 95% CI 1.01–1.03, P < 0.001), as presented in Table 2 and Figure 3.

**Table 2:** Univariate logistics and multivariate logistics regression analysis for risk factors in CKD of the January 2018 to December 2023 cohort.

Variables	Univariate					Multivariate					VIF
	**β**	**S.E**	**t**	***P***	**OR (95%CI)**	**β**	**S.E**	**t**	***P***	**OR (95%CI)**	
Gender											
Male					Male (Reference)					Male (Reference)	
Female	−0.15	0.26	−0.59	0.554	0.86 (0.52 ~ 1.42)	−0.11	0.32	−0.33	0.740	0.90 (0.48 ~ 1.68)	1.077
Age (year)	0.03	0.01	2.99	0.003	1.03 (1.01 ~ 1.05)	0.03	0.01	2.35	0.019	1.03 (1.01 ~ 1.05)	1.076
DBP (mmHg)	−0.02	0.01	−1.67	0.095	0.98 (0.95 ~ 1.00)	−0.01	0.02	−0.86	0.388	0.99 (0.95 ~ 1.02)	1.061
HDL-C (mmol/L)	−1.18	0.43	−2.73	0.006	0.31 (0.13 ~ 0.72)	−0.39	0.53	−0.73	0.466	0.68 (0.24 ~ 1.92)	1.125
BUN (mmol/L)	0.40	0.04	10.14	<.001	1.49 (1.38 ~ 1.61)	0.16	0.05	3.37	<.001	1.17 (1.07 ~ 1.29)	3.072
Scr (μmol/L)	0.03	0.00	9.71	<.001	1.03 (1.02 ~ 1.03)	0.01	0.00	4.84	<.001	1.01 (1.01 ~ 1.02)	3.592
Serum lipase (U/L)	0.04	0.00	9.24	<.001	1.04 (1.03 ~ 1.05)	0.02	0.00	4.15	<.001	1.02 (1.01 ~ 1.03)	2.276

**Note:** OR: Odds Ratio, CI: Confidence Interval. DBP: diastolic blood pressure; HDL-C: high-density lipoprotein cholesterol; Scr: serum creatinine; BUN: blood urea nitrogen; VIF: variance inflation factor; β: regression coefficients; S.E: standard errors; model fitting information (χ^2^ = 279.141, P < 0.001); parallel lines test (χ^2^ = 22.923, P = 0.062).

### 3.5 Diagnostic performance and validation of LPS across CKD stages

**Fig 4:**
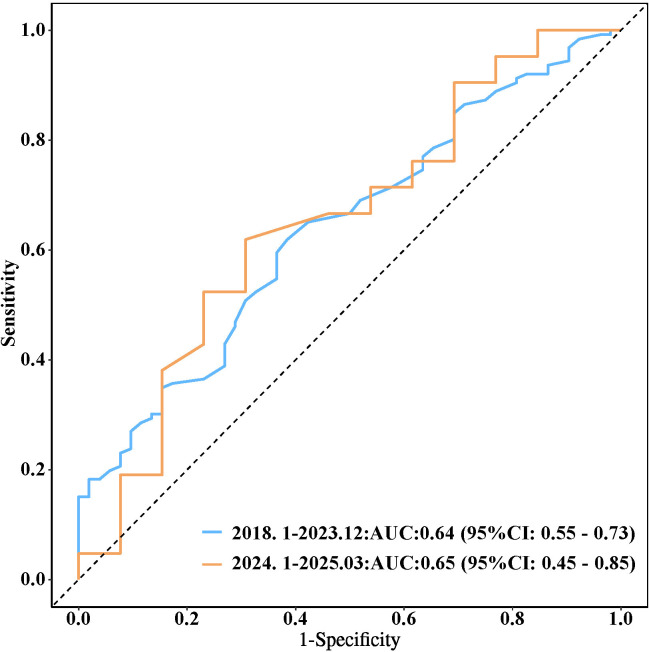
Receiver Operating Characteristic curves for serum lipase predictive score in CKD stages G1-2 and G3. Note: ROC curves for two cohorts are displayed: training cohort patients from Jan 2018 to Dec 2023 with AUC = 0.64 (95% CI: 0.55–0.73) and validation cohort patients from Jan 2024 to Mar 2025 with AUC = 0.65 (95% CI: 0.45–0.85).

**Fig 5:**
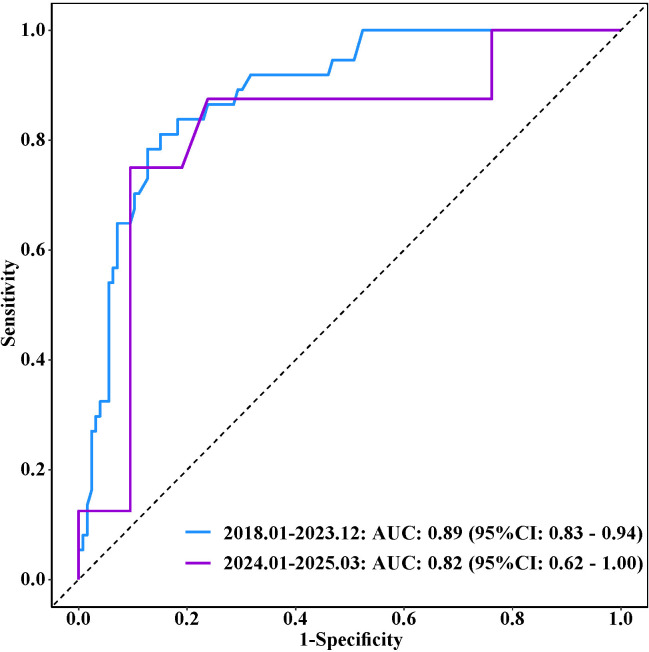
Receiver Operating Characteristic curves for serum lipase predictive score in CKD stages G3 and G4. Note: ROC curves for two cohorts are displayed: training cohort patients from Jan 2018 to Dec 2023 with AUC = 0.89 (95% CI: 0.83–0.94); and validation cohort patients from Jan 2024 to Mar 2025 with AUC = 0.82 (95% CI: 0.62–1.00).

**Fig 6:**
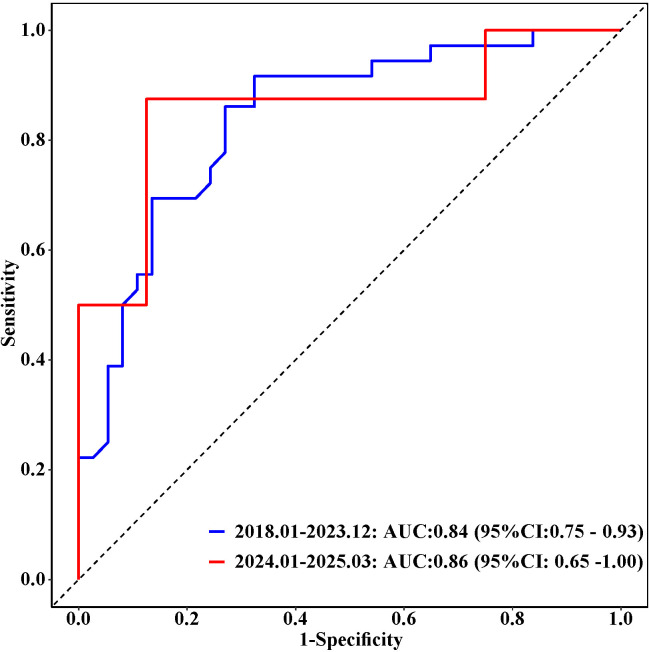
Receiver Operating Characteristic curves for serum lipase predictive score in CKD stages G4 and G5. Note: ROC curves for two cohorts are displayed: training cohort patients from Jan 2018 to Dec 2023 with AUC = 0.84, 95% CI: 0.75–0.93) and validation cohort patients from Jan 2024 to Mar 2025 with AUC = 0.86, 95% CI: 0.65–1.00).

The analysis of the Receiver Operating Characteristic (ROC) curve demonstrated the predictive efficacy of serum lipase (LPS) in distinguishing between different stages of chronic kidney disease (CKD) within the training cohort. Specifically, LPS achieved area under the curve (AUC) values of 0.64 (95% confidence interval: 0.55–0.73) for differentiating stages G1–G2 from G3, 0.89 (95% CI: 0.83–0.94) for G3 from G4, and 0.84 (95% CI: 0.75–0.93) for G4 from G5. The corresponding sensitivities were 62%, 85%, and 68%, while the specificities were 62%, 81%, and 92%, respectively, as detailed in Table 3. External validation, conducted with 50 CKD cases prospectively enrolled from January 2024 to March 2025 [note: future recruitment period], yielded similar AUC values of 0.65 (95% CI: 0.45–0.85), 0.82 (95% CI: 0.62–1.00), and 0.86 (95% CI: 0.65–1.00), as illustrated in Figs 4–6.

**Table 3:** Predictive value of LPS for CKD stage of the January 2018 to December 2023 cohort.

CKD stage	Cut off value of LPS(U/L)	AUC (95%CI)	Accuracy (95%CI)	Sensitivity (95%CI)	Specificity (95%CI)	PPV (95%CI)	NPV (95%CI)
G 1–2 versus G 3	42.5	0.64 (0.55-0.73)	0.62 (0.54-0.69)	0.62 (0.48 - 0.75)	0.62 (0.53 - 0.70)	0.40 (0.29 - 0.51)	0.80 (0.72 - 0.88)
G 3 versus G 4	71.0	0.89 (0.83-0.94)	0.84 (0.78-0.89)	0.85 (0.79 - 0.91)	0.81 (0.68 - 0.94)	0.94 (0.89 - 0.98)	0.61 (0.48 - 0.75)
G 4 versus G 5	108.0	0.84 (0.75-0.93)	0.79 (0.68-0.88)	0.68 (0.52 - 0.83)	0.92 (0.83 - 1.00)	0.89 (0.78 - 1.00)	0.73 (0.60 - 0.86)

Abbreviations: CKD: chronic kidney disease; LPS: serum lipase; AUC: area under the curve; PPV: positive predictive value; NPV: negative predictive value; CI: confidence interval.

## 4. Discussion

Chronic kidney disease (CKD) is characterized by damage to the renal tubules and glomeruli, typically manifesting as a progressive decline in glomerular filtration rate (GFR), which may ultimately lead to end-stage renal disease requiring renal replacement therapy. CKD detrimentally impacts patients’ quality of life and is strongly associated with an increased risk of cardiovascular disease, as well as substantial healthcare costs [17]. While previous studies have reported abnormal serum lipase levels in patients with CKD, the existing literature has primarily focused on static assessments and isolated observational analyses. This study introduces a novel approach by examining serum lipase levels across various stages of CKD. Serum lipase was identified as an associated factor in a cross-sectional analysis, and these findings were subsequently validated in an independent CKD cohort. To further elucidate these implications, this study undertakes a retrospective analysis of baseline characteristics, biomarkers, and clinical data from 251 patients with chronic kidney disease (CKD), investigating the association between serum lipase levels and CKD stages. The findings of this research provide offer a foundational basis for the development of future monitoring and treatment strategies.

The analysis of serum lipase levels in patients with different stages of chronic kidney disease (CKD) revealed a positive correlation between increased lipase levels and the progression of CKD severity, highlighting a significant association between elevated serum lipase levels and disease advancement. These findings indicate that serum lipase could serve as a valuable biomarker for assessing CKD status and monitoring treatment efficacy. Although this study supports the trend observed by Regasa T et al. [18], the observed differences in serum lipase levels across stages may be attributed to variations in the study populations. Regasa T’s research focused on diabetic patients in stages G1 to G4, whereas the present study included patients across all CKD stages from G1 to G5. The role of lipase in chronic kidney disease (CKD) is intricate and multifaceted, potentially contributing to disease progression through various mechanisms. Li X et al. identified lipid peroxidation as a pivotal factor in the transition from acute kidney injury (AKI) to CKD, noting that lipase activity may influence renal fibrosis by modulating lipid metabolism [19]. These findings imply that lipase may directly exacerbate renal injury by promoting lipid peroxidation and the release of pro-inflammatory cytokines. Furthermore, research by Tramontano D et al. revealed a significant reduction in lipoprotein lipase levels in patients with CKD, suggesting that lipase dysfunction may contribute to abnormal lipid deposition within the kidneys, thereby activating oxidative stress and inflammatory pathways, and participating in CKD pathogenesis [20]. However, these compensatory mechanisms may not always be effective and could potentially worsen renal injury [21]. Further investigation is required to elucidate the clinical and therapeutic implications of these mechanisms.

This study undertook a comprehensive examination of the relationship between serum lipase and key renal function indicators, specifically serum creatinine (Scr) and blood urea nitrogen (BUN). The analysis revealed positive correlations between serum lipase levels and both Scr and BUN. These findings suggest that serum lipase may play a role in the progression of chronic kidney disease (CKD) and could potentially serve as a biomarker for evaluating the severity of renal impairment. Notably, the correlation coefficients between serum lipase and Scr/BUN observed in this study surpassed those reported by Khan SI et al. [22], which may be due to their focus on dialysis patients with more advanced clinical conditions. Previous research has predominantly emphasized lipid metabolism disorders in CKD [23] and their effects on renal function, including the roles of inflammatory factors and metabolites [24], with limited attention given to changes in lipase levels and their clinical significance. The observed correlation between serum lipase levels and the progression of CKD underscores the need for further investigation into its pathophysiological implications. Our findings demonstrate a significant correlation between elevated lipase levels and renal dysfunction, as evidenced by a correlation coefficient of r = 0.714 with serum creatinine. However, the observational design of the study precludes definitive causal inferences. The observed elevation in lipase levels may represent either a compensatory mechanism or a direct contributor to lipid-mediated inflammatory nephrotoxicity. These results offer valuable insights for further exploration of the mechanistic role of serum lipase in chronic kidney disease (CKD). They imply that monitoring lipase levels could enhance the assessment of disease progression and inform personalized treatment strategies for patients with CKD. Nonetheless, further research is imperative to elucidate the specific mechanisms and clinical utility of serum lipase in CKD. Additionally, it is essential to investigate interventions aimed at reducing lipase levels, such as lipid-lowering agents or dietary modifications, to evaluate their potential impact on CKD outcomes. Future prospective studies should prioritize assessing the therapeutic benefits of these strategies within CKD populations. Additionally, the integration of routine serum lipase monitoring into CKD management protocols, especially for high-risk patients, merits consideration.

This study elucidates that serum creatinine (Scr), blood urea nitrogen (BUN), and serum lipase independently predict the progression of chronic kidney disease (CKD), as demonstrated through both univariate and multivariate logistic regression analyses. The findings reinforce the significance of traditional biomarkers (Scr, BUN, and eGFR) in assessing renal function, while highlighting the emerging significance of serum lipase as a novel biomarker for CKD stages. Serum lipase, a pancreatic enzyme, influences renal health through mechanisms involving lipid metabolism, inflammation, and the interaction between pancreatic and renal functions. This research distinguishes itself from prior studies that focused on a single or limited set of factors by utilizing a comprehensive array of variables to construct the regression model, thereby enhancing the validity and reliability of the results through rigorous statistical methodologies. Serum lipase demonstrated robust diagnostic performance across various stages of chronic kidney disease (CKD), with the highest discriminatory capability observed in distinguishing stage G3 from G4. However, its effectiveness was comparatively lower in differentiating stages G1–G2 from G3. Validation conducted with 50 CKD cases enrolled from January 2024 to March 2025 yielded consistent results, reinforcing the clinical utility of LPS for CKD staging and prognostic stratification. To mitigate the potential confounding effects of severe hypertriglyceridemia, which can artifactually reduce lipase measurements, lipemic serum samples were subjected to ultracentrifugation prior to analysis. This approach aligns with recommendations for minimizing pre-analytical variability in biomarker studies. Although triglyceride (TG) levels did not significantly differ across CKD stages (P > 0.05) in our cohort, this precaution remains essential given the high prevalence of dyslipidemia in CKD populations. Although our findings indicate a stage-dependent increase in serum lipase during the progression of chronic kidney disease (CKD), its ability to discriminate early stages remains limited. The area under the curve (AUC) of 0.64 for distinguishing between stages G1-2 and G3 suggests only modest diagnostic performance, which is insufficient to recommend its routine clinical use in early CKD detection. Novel biomarkers often achieve optimal performance when incorporated into multi-biomarker panels rather than when used in isolation. Until validated through larger prospective studies and potentially integrated with other emerging biomarkers, lipase should not be considered a standalone screening tool for the early stages of CKD.

While this study provides valuable insights into the potential role of serum lipase in the staging of chronic kidney disease (CKD), it is crucial to acknowledge several limitations. Firstly, the lack of comprehensive data on other pancreatic and extra-pancreatic enzymes, such as amylase, trypsin, elastase-1, and phospholipase A2, restricts the ability to perform a comparative analysis of the diagnostic performance of lipase relative to other enzymatic biomarkers. Such an analysis could further elucidate the prognostic value of these biomarkers in CKD. Secondly, the relatively small sample size may introduce bias, and the absence of repeated measurements of relevant indicators in patients could lead to data inaccuracies, potentially hindering a thorough understanding of CKD stages. Thirdly, although acute pancreatic disorders and acute kidney injury were excluded from the study, unmeasured confounding variables, such as dietary habits, medication use, alcohol consumption below the exclusion threshold, and individual metabolic variability, may still influence the observed associations. Future research should therefore prioritize the execution of large-scale, longitudinal studies that include diverse patient populations, integrate additional biomarker assessments, and rigorously control for confounding variables to validate and enhance the clinical utility of serum lipase as a factor associated with the progression of chronic kidney disease (CKD). Prospective cohort studies are needed to investigate the temporal relationships between elevated lipase levels and renal functional decline. Additionally, mechanistic studies employing CKD animal models are crucial to determine whether lipase plays a direct role in interstitial fibrosis or merely coexists with it.

## 5. Conclusion

Serum lipase demonstrates potential as an associated factor in the progression of chronic kidney disease (CKD), particularly in its advanced stages. However, its efficacy as a standalone marker for early detection is inadequate to warrant routine clinical use. Future research should aim to validate the inclusion of lipase in multi-biomarker panels and to delineate its optimal role within CKD risk stratification protocols. The prospective clinical utility of integrating this readily measurable parameter into existing CKD risk models requires validation through multicenter prospective trials. Our findings suggest that regular monitoring of lipase levels could improve the assessment of therapeutic outcomes and prognostic stratification when used alongside conventional biomarkers. Future investigations should prioritize longitudinal cohort studies to establish causality and explore whether interventions targeting lipid metabolism pathways can slow disease progression in CKD patients with elevated lipase levels. Such integrative strategies may enhance personalized management approaches in CKD care.

## Supporting information

S1 Data (XLS)
